# What is the best management option for non-significant residual shunt after device closure of perimembranous ventricular septal defect: A case report based on the lessons from post-procedure endocarditis

**DOI:** 10.1097/MD.0000000000017347

**Published:** 2019-10-18

**Authors:** Shuran Shao, Chunyan Luo, Kaiyu Zhou, Yimin Hua, Chuan Wang

**Affiliations:** a Department of Pediatric Cardiology, West China Second University Hospital, Sichuan University; b Department of Radiology, West China Hospital, Sichuan University; c The Cardiac Development and Early Intervention Unit, West China Institute of Women and Children's Health, West China Second University Hospital, Sichuan University; d Key Laboratory of Birth Defects and Related Diseases of Women and Children (Sichuan University), Ministry of Education Chengdu; e Key Laboratory of Development and Diseases of Women and Children of Sichuan Province, West China Second University Hospital, Sichuan University, Chengdu, Sichuan, China.

**Keywords:** infective endocarditis, residual shunt, ventricular septal defect

## Abstract

**Rationale:**

Non-significant residual shunt is a relatively common complication after device closure of perimembranous ventricular septal defects (Pm-VSD). Lifelong antibiotic prophylaxis has been recommended in guidelines to avoid infectious endocarditis (IE) if residual shunt remains. Clinicians, however, rarely follow it in their practice and regular follow-up was the most common option since post-procedure IE after transcatheter closure of PmVSD is rarely reported. We firstly described a case of IE after transcatheter closure of PmVSD with modified symmetrical double-disk device with a residual shunt, highlighting the need for reassessing the prognostic implications of post-procedure non-significant residual shunt and the most appropriate treatment strategy.

**Patient concerns:**

A 3-year old female received transcatheter closure of PmVSD sized 5.0 mm on left ventricular angiography with an 8-mm modified symmetric double-disk occluder (SHAMA) owing to a history of recurrent lower respiratory tract infections. Post-procedure echocardiography documented a non-significant residual shunt, but no additional interventions were performed. Two months post procedure, the child was re-admitted into our department with a complaint of persistent fever up to 41°C for 11 days and nonresponse to 1-week course of amoxicillin.

**Diagnoses:**

The diagnosis of post procedure IE was established since a vegetation (13 × 9 mm) was found to be attached to the tricuspid valve and the occluder, and *Staphylococcus aureus* was isolated from all three-blood cultures.

**Interventions:**

After 6 weeks of vancomycin treatment, the vegetation disappeared with no sign of valvular dysfunction. Three weeks after discharge, a second device was implanted to abolish persistent residual flow.

**Outcomes:**

Unfortunately, the child was ultimately transferred to surgical department due to severe hemolysis after the second device implantation. The occluders were removed and the VSD was closed with a pericardial patch. Tricuspid valvuloplasty was also performed and the following course was uneventful.

**Lessons:**

For non-significant residual shunt after device closure of PmVSD, implantation of a second device or surgical repair may be a better and more satisfactory alternative compared with lifelong antibiotic prophylaxis or no interventions, since associated IE can indeed occur despite its rarity and the risk of antibiotic-associated adverse events may outweigh the benefits.

## 1 Introduction

Transcatheter closure of perimembranous ventricular septal defect (PmVSD) has been proved to be a safe and effective alternative to surgery in selected patients.^[1,2]^ Residual shunt is recognized as a relatively common complication post procedure.^[1]^ However, less concern and attention are raised from cardiologists for long-term prognostic implications of post procedure residual shunt since most of them occurred early and a high proportion are temporary and non-significant. Theoretically, residual shunt could increase risk for post-interventional IE via endothelial damage resulting from mechanical lesions provoked by turbulent blood flow across the defect. Indeed, lifelong antibiotic prophylaxis has been recommended for any type of congenital heart defect repaired with a prosthetic material, whether placed surgically or by percutaneous techniques if residual shunt remains.^[3,4]^ Clinicians, however, rarely follow the current guidelines in their practice.^[5]^ Post-procedure IE after transcatheter closure of PmVSD is rarely reported.^[6,7]^ Herein, we first described a case of IE after transcatheter closure of PmVSD with modified symmetrical double-disk device with a residual shunt, highlighting the need for reassessing the prognostic implications of post procedure non-significant residual shunt and the most appropriate treatment strategy.

## 2 Ethics statements

Informed written consent was obtained from the parents after the nature of this study had been fully explained to them. The parents of patient have provided informed consent for publication of the case.

## 3 Case report

**Figure 1:**
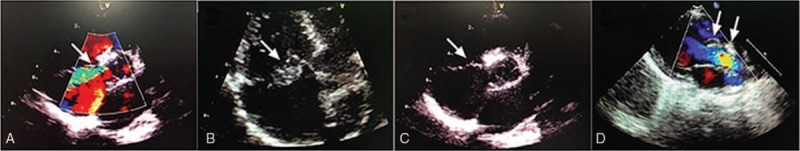
Findings on transthoracic echocardiography (TTE). (A) Parasternal short-axis view showing the residual shunt (white arrow) after procedure. (B) Four-chamber view showing vegetation attached to tricuspid valve (white arrow). (C) Parasternal short-axis view revealing disappearance of vegetation following antibiotic therapy. (D) Parasternal long-axis view revealing no residual shunt after implantation of another occluder (white arrow).

A 3-year old female weighing 10.5 Kg, with a PmVSD and a history of recurrent lower respiratory tract infections, was referred to our hospital for transcatheter closure of the defect. Informed consent to the procedure was obtained from the child's parents. The procedure was undertaken under general anesthesia and performed in a standard way detailed in our previous study.^[8]^ The defect measured 5.0 mm on left ventricular angiography and an 8-mm modified symmetric double-disk occluder (SHAMA) was chosen. The device was released despite a small residual shunt was noted immediately after the procedure. Oral administration of aspirin (50 mg daily) was initiated and the child was subjected to 72 hours of dynamic ECG monitoring, as well as a 12-lead ECG and echocardiography at 1, 3, 7 days post procedure, during which time the patient was uneventful except for the residual shunt (Fig. 1A) and discharged 1 week later.

Two months after the procedure, the child was re-admitted into our department with a complaint of persistent fever up to 41°C for 11 days and nonresponse to 1-week course of amoxicillin. On admission, the child was conscious with temperature of 38.7°C, heart rate of 110 per minute, respiratory rate was 30 per minute, and blood pressure was 96/50 mmHg. Physical examination was only remarkable for a 2/6 systolic murmur at the second and third left intercostal space. Laboratory tests revealed an elevated white blood cell count of 16.8 × 10^9^/L, neutrophil percentage of 62.7%, C-reactive protein of 75.0 mg/L, erythrocyte sedimentation rate of 32.0 mm/h, and mild anemia (hemoglobin: 105 g/L). Transthoracic echocardiography (TTE) demonstrated a vegetation (13 × 9 mm) attached to tricuspid valve and the occluder, and a small residual shunt (Fig. 1B). Three sets of blood cultures were taken and empiric antibiotic therapy consisting of vancomycin and ceftriaxone was initiated. *Staphylococcus aureus* was isolated from all 3-blood cultures. Treatment with vancomycin was continued since the isolates were resistant to penicillin but sensitive to vancomycin. The origin of this infection was not found. Results of a dental checkup performed during hospitalization were normal. Defervescence occurred in 20 days following dosage adjustment of vancomycin. After 6 weeks of treatment, the vegetation disappeared with no sign of valvular dysfunction (Fig. 1C), and thereafter the child was discharged. Three weeks later, a second device was implanted to abolish persistent residual flow. Unfortunately, the child was ultimately transferred to surgical department due to severe hemolysis after the second device implantation. During the surgical procedure, the occluders were removed and the VSD was closed with a pericardial patch. Additionally, tricuspid valvuloplasty was also performed and the following course was uneventful.

## 4 Discussion

Even though native VSD endocarditis is well known,^[9]^ device-associated endocarditis following PmVSD closure is scarce. Up to now, only 2 cases with post procedure IE occurred 10 days^[7]^ and 4 months^[6]^ after transcatheter closure of PmVSD with Nit-Occlud Le device, respectively, have been reported. To our knowledge, this is the first case of IE complicated by residual shunt after percutaneous PmVSD occlusion using modified symmetric double-disk occluder. A recent meta-analysis documented that residual shunt is the most common complication following device closure of PmVSD, with a pooled rate of 15.9%.^[1]^ The present report was of clinically amount significance for illustrating the need to reassess the prognostic implications of non-significant residual shunt after PmVSD device closure and consider the most reasonable therapeutic regimen.

Lifelong antibiotic prophylaxis has been recommended for any type of congenital heart defect repaired with a prosthetic material, whether placed surgically or by percutaneous techniques if residual shunt remains.^[4,10]^ However, it appears to be radical and difficult for clinicians to follow the guidelines^[5]^ since post-procedure IE after transcatheter closure of PmVSD, even with residual shunt, are rarely reported, most importantly, the risk of antibiotic-associated adverse events may outweigh the benefits and a very small number of cases may be prevented by prophylaxis. On the other hand, no interventions and merely regular follow up may also not be recommended since the associated IE can indeed occur despite its rarity. Alternatively, implantation of a second device or surgical repair could be considered and it may be a better and more satisfactory means for abolishing persistent residual flow and preventing endothelial damage resulting from mechanical lesions provoked by turbulent blood flow across the defect, despite the risk of endocarditis require further evaluation.

In conclusion, for non-significant residual shunt after device closure of PmVSD, implantation of a second device or surgical repair may be a better and more satisfactory alternative compared with lifelong antibiotic prophylaxis or no interventions, since associated IE can indeed occur despite its rarity and the risk of antibiotic-associated adverse events may outweigh the benefits.

## Author contributions

**Resources:** Chunyan Luo, Chuan Wang.

**Supervision:** Kaiyu Zhou, Yimin Hua, Chuan Wang.

**Writing – original draft:** Shuran Shao.

**Writing – review & editing:** Chunyan Luo, Chuan Wang.
